# Overview of Cochrane Systematic Reviews of Rehabilitation Interventions for Persons with Traumatic Brain Injury: A Mapping Synthesis

**DOI:** 10.3390/jcm11102691

**Published:** 2022-05-10

**Authors:** Vanessa M. Young, Juan R. Hill, Michele Patrini, Stefano Negrini, Chiara Arienti

**Affiliations:** 1 School of Social and Behavioral Sciences, Arizona State University, Phoenix, AZ 85051, USA; vmyoung1@asu.edu; 2 Independent Researcher, San Diego, CA 92108, USA; ricardo2hill@gmail.com; 3 Laboratory of Evidence-Based Rehabilitation, IRCCS Istituto Ortopedico Galeazzi, 20161 Milan, Italy; mikepatrini@gmail.com; 4 Department of Biomedical, Surgical and Dental Sciences, University La Statale, 20122 Milan, Italy; 5 IRCCS Fondazione Don Carlo Gnocchi, 20148 Milan, Italy; carienti@dongnocchi.it

**Keywords:** brain injuries, traumatic, interventions, treatment outcome, rehabilitation, overview

## Abstract

Background: The World Health Organization has identified an unmet global need for rehabilitation interventions concerning 20 non-communicable diseases, traumatic brain injury included. This overview compiles and synthesizes the quality and quantity of available evidence on the effectiveness of rehabilitation interventions for traumatic brain injury from Cochrane systematic reviews (CSRs). The results will be used to develop the Package of Interventions for Rehabilitation. Methods: All CSRs on TBI tagged in the Cochrane Rehabilitation database published between August 2009 and September 2021 were included. Evidence mapping was implemented to extract study characteristics and evidence from the CSRs. Results: Six CSRs (42 studies; *n* = 3983) examined the effectiveness of either non-pharmacological or pharmacological interventions after TBI. Among 19 comparisons, 3% were rated as high in quality of evidence, 9% moderate, 54% low, and 34% very low. Non-pharmacological interventions with moderate quality, hospital-based cognitive rehabilitation and cognitive didactic therapy, likely produced minimal to no changes in the return-to-work rate. Anti-epileptic drugs and neuroprotective agents resulted in a minimal difference to the frequency of late seizure episodes in post-traumatic epilepsy. Conclusions: No prominent advances in treatment options were reported in any of the CSRs. The high rate of low and very low quality of evidence makes it difficult to ascertain the effectiveness of several recommended non-pharmacological interventions.

## 1. Introduction

The World Health Organization (WHO) has described an unmet global need for the delivery of rehabilitation interventions in health systems, which is amplified in low- and middle- income countries with limited availability of resources [1,2,3]. The ‘WHO Rehabilitation 2030 Call for Action’ [2] was therefore launched. One of the main actions considered is the development of a Package of Interventions for Rehabilitation (PIR) [3,4]. The PIR aims at promoting favorable outcomes, accessibility, and the integration of multidisciplinary/interdisciplinary rehabilitation services into healthcare systems worldwide [3,4]. The WHO identified 20 major noncommunicable diseases to be investigated to develop the PIR; among these is traumatic brain injury (TBI) [4].

TBI is defined as ‘any alteration in brain function or other evidence of brain pathology caused by an external force’ [5] and it is estimated to affect 69,000 individuals worldwide annually [6]. Alterations in brain function may include any of the following: loss of (or decrease in) consciousness; loss of memory of events immediately preceding or following the injury; neurologic deficits (e.g., loss of balance or vision); or altered mental status, such as disorientation or confusion at the time of the injury [5]. TBI can be categorized into three possible diagnostic levels (mild, moderate, or severe), typically after evaluation using the Glasgow Outcome Scale or Glasgow Outcome Scale Extended [7,8] or by assessing structural imaging, loss of consciousness, altered consciousness, or post-traumatic amnesia.

Research has identified falls and road injuries as the two main causes of TBI worldwide [9,10], although causes of TBI have been found to differ across countries, depending on income, geographical region, and political circumstances [9,11]. Other common causes include sports-related concussions, assault, interpersonal violence, and blast injuries [12]. The direct consequences of a single TBI or repetitive insults include many possible long-term sequelae that vary according to age, sex, and the nature of the injury [13,14]. Common secondary pathophysiological conditions include seizures, sleep disorders, neurodegenerative diseases, neuroendocrine dysregulation, and psychiatric issues, each of which may persist throughout the long-term recovery process following moderate-to-severe TBI [15]. Due to these numerous clinical and demographic variables, TBI patients often experience nonlinear recovery trends, and those with moderate and severe cases are reported to show deteriorating Glasgow Outcome Scale Extended scores over time [16]. These unfavorable outcomes can hinder functioning, quality of life, and employment, and may worsen pre-existing conditions [17], further highlighting the chronic health issues associated with TBI as well as the need for complex rehabilitative programs and long-term services to support this group of patients [16].

A major step to the development of the PIR encompasses the “Best Evidence for Rehabilitation” (be4rehab) approach, which is applied to this work. Be4rehab supports the gathering of best evidence on the effectiveness and quality of pharmacological and non-pharmacological rehabilitation interventions for individuals with TBI and the delivery of this overview of Cochrane systematic reviews (CSRs) [4]. Overviews of systematics reviews are a methodological approach proposed by Cochrane to compile and synthesize data from multiple systematic reviews into one single, accessible document. All overviews requested by the WHO are restricted to CSRs to preserve the coherence and quality of the gathered evidence. 

Supplemented by evidence mapping to aid in the synthesis of available evidence, this work aims at identifying the broad quality and the quantity of evidence, published in CSRs, on the effectiveness of rehabilitation interventions in person with TBI.

## 2. Materials and Methods

The WHO PIR adheres to methods designed from the collaborative efforts of the WHO Rehabilitation Programme and Cochrane Rehabilitation, and the directives from the WHO Guidelines Review Committee [4]. We used evidence mapping to synthesize and visualize study characteristics and evidence from CSRs on TBI. The overview was registered in Open Science Framework Registries (https://doi.org/0.17605/OSF.IO/M5XVG) and was reported following the Preferred Reporting Items for Systematic Reviews and Meta-analysis (PRISMA 2020 statement) [18].

### 2.1. Search Strategy

According to the methodology developed by the Cochrane Rehabilitation [19], CSRs relevant to rehabilitation are continuously tagged to maintain an up-to-date database (https://rehabilitation.cochrane.org/evidence, accessed on 1 September 2019). We initially searched all CSRs related to TBI published between August 2009 and August 2019 and reported the results to the WHO. We subsequently searched the Cochrane Library to August 2021 to preserve the timeliness of evidence. Eligible CSRs included those assessing interventions for persons with TBI provided or prescribed by rehabilitation professionals [19].

We included only tagged CSRs that examined rehabilitation interventions on individuals with TBI, of any age and gender. CSRs focused on persons with acquired brain injury or non-traumatic brain injury were excluded to ensure that the evidence synthesis is strictly applicable to persons who sustained a TBI.

### 2.2. Assessment of Methodological Quality of Included Studies

The methodological quality of each CSR was appraised by two assessors using the 16-item A Measurement Tool to Assess Systematic Reviews (AMSTAR) 2 tool. In this updated version, the 16 items are scored on a binary yes or no scale. AMSTAR-2 does not generate an ‘overall score’; a high score may disguise weaknesses in 7 critical items [20]. The assessors adopted a process of ‘considered judgment’, which entails (1) interpreting weaknesses detected by the critical items and (2) reaching a consensus on the methodological quality of each CSR. Disagreements were resolved through discussion with a third assessor.

### 2.3. Data Extraction and Quality of Evidence Appraisal

The authors referred to the Table of Findings presented in each of the included CSRs; these contain the following data: type of outcome, outcome measure(s), number of primary studies, sample sizes, type of population, intervention, comparator(s), and effect (i.e., no effect, in favor of intervention, or in favor of comparator). Data were collected and entered into an Excel datasheet.

In addition, the quality of evidence for each outcome was extracted using the Grading of Recommendations Assessment, Development, and Evaluation (GRADE) rating system. For CSRs that did not include GRADE ratings, two members of the Cochrane Rehabilitation team independently appraised the quality of evidence for the primary outcomes only using the GRADE approach [21]. Any disagreement was resolved through consensus decision-making involving a third author [22]. The GRADE appraisal approach included two steps: (1) retrieval of the original primary studies included in each CSR; and (2) tabulation of the quality of evidence provided in Summary of Findings tables using GRADEPro software.

### 2.4. Summarizing the Data with an Evidence Map

Quality of evidence and effect data were transferred into evidence maps developed in Excel. The evidence map integrates the outcome and rehabilitation intervention values for each comparison. The magnitude of the effect (i.e., no effect, in favor of intervention, or in favor of comparator) and the quality of evidence (i.e., very low, low, moderate, or high) were presented laterally and color-coded for each outcome in order to generate a visual aid to facilitate the understanding of the overall judgement of the evidence.

Evidence mapping was employed as a complementary method to collating and appraising evidence from the CSRs, and subsequently used to summarize the results for the overview. The instrument collated outcomes and rehabilitation interventions and resulted in a comprehensive overview of the quality of evidence and effects. Because we did not consider other outcomes and interventions in addition to the those examined in the included CSRs, evidence mapping was not used to identify evidence gaps.

## 3. Results

**Figure 1:**
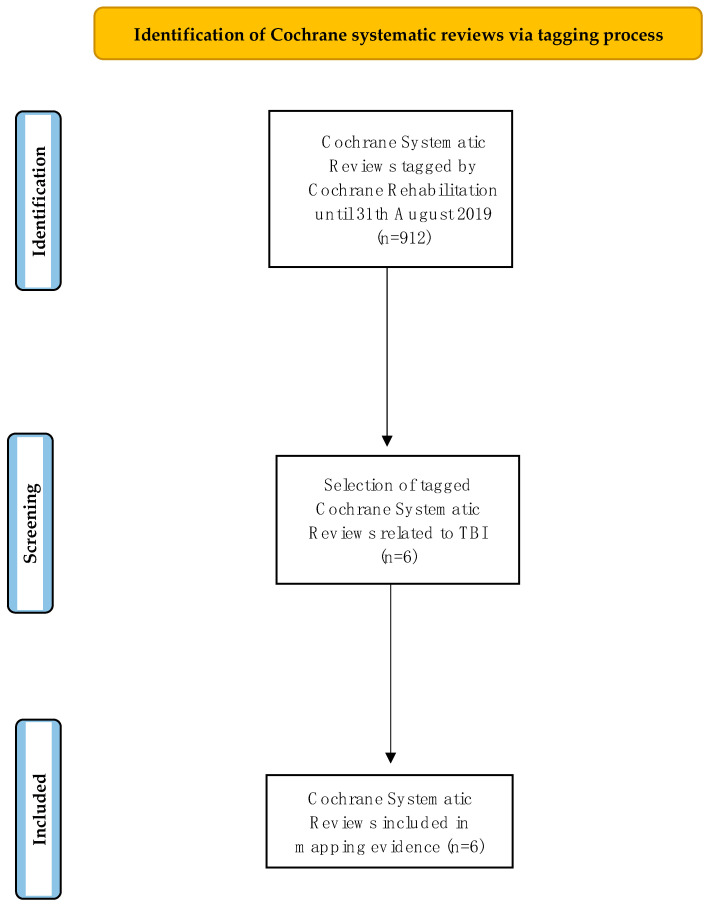
Flow chart displaying the tagging process of Cochrane systematic review.

The authors identified six tagged CSRs related to TBI: one published in 2013 [23], two in 2015 [24,25], and three in 2017 [26,27,28] (see Figure 1).

Three CSRs included only participants who sustained a TBI and excluded people with acquired brain injury and non-traumatic injury. Two CSRs included studies with a mixed population only when disaggregated data were reported to ensure that evidence was relevant to TBI. Finally, one CSR reported including studies where the etiology of the TBI is uncertain. The characteristics of the included systematic reviews are reported in Table 1.

Comprehensively, this mapping review encompasses 42 primary studies, 3983 participants, and 19 comparisons that examined the effectiveness and safety of either non-pharmacological or pharmacological interventions for individuals with TBI. Among non-pharmacological comparisons, four interventions (six outcomes) were categorized as very low quality of evidence, and eight interventions (16 outcomes) were deemed as low quality of evidence. Among the pharmacological comparisons, we found that four interventions (six outcomes) were rated very low and three interventions (three outcomes) were rated low in quality. The AMSTAR 2 assessment tool identified high methodological quality in the six CSRs; even when sources of funding were not reported. Results of the AMSTAR 2 assessment are displayed in Table 2.

The evidence map findings were divided into two categories: (1) non-pharmacological interventions and (2) pharmacological interventions. Table 3 provides an overview of evidence map finding for non-pharmacological interventions for TBI. Table 4 provides an overview of evidence map finding for pharmacological interventions for TBI.

### 3.1. Quality of Evidence Mapping for Non-Pharmacological Interventions

#### 3.1.1. Moderate Quality of Evidence

Hospital-based versus home-based cognitive rehabilitation likely has little to no effect on the return-to-work rate for moderate-to-severe TBI (1 study; *n* = 120) [26]. Similarly, cognitive didactic versus functional experiential therapy likely has little to no effect on the same outcome for moderate-to-severe TBI (1 study, *n* = 366) [26].

#### 3.1.2. Low Quality of Evidence

Exercise using large muscle groups may have little to no effect on the cardiorespiratory fitness compared to usual care in severe and unspecified TBI severity levels (3 studies, *n* = 67) [27].

Cognitive rehabilitation may have little or no effect compared to no treatment on community integration in severe TBI (1 study; *n* = 12) [26], while it may have little to no effect relative to conventional therapy on return to work (1 study; *n* = 68) [26], and community integration (3 studies; *n* = 123) [26] in mild-to-severe TBI, respectively.

Electro-acupuncture as an adjunct treatment to rehabilitation training may have a positive effect on sensorimotor impairment (Fugl-Meyer Assessment) at 1 and 3 months, and on disability (Modified Barthel index) at 1 month, but not at 3 months, when the effects favored rehabilitation training alone (unspecified TBI severity; 1 study; *n* = 150) [23]. When added to conventional medical intervention, electro-acupuncture may make little to no difference to mortality rate, but it may increase the frequency of normal Glasgow Coma Score evaluations in coma patients with severe TBI (1 study, *n* = 50) [23]. Added to hyperbaric oxygen and rehabilitation training, electro-acupuncture may have an effect on the percentage of patients decreasing to moderate disability (Barthel Index > 60) but there is uncertainty on the effects on reducing its severity (Barthel Index > 40) (unspecified TBI severity; 1 study; *n* = 122) [23].

#### 3.1.3. Very Low Quality of Evidence

In mild-to-moderate TBI, the true effect of cognitive rehabilitation remains uncertain on return-to-work when compared to no treatment (1 study; *n* = 50) [26]; on activities of daily living when compared to conventional therapy (unspecified TBI severity; 2 studies, *n* = 41) [26]; on depression level versus waiting list (3 studies, *n* = 146) [24] and supportive psychotherapy (1 study; *n* = 48) [24]. There is also uncertainty on the utility on spasticity (6 h post-treatment) of repositioning splints equipped with participant-specific pseudoelastic hinges versus traditional splints with fixed angle braces for pediatric TBI (unspecified TBI severity; 1 study; *n* = 25) [28].

### 3.2. Quality of Evidence Mapping for Pharmacological Interventions

#### 3.2.1. High Quality of Evidence

Neuroprotective agents had little to no effect versus placebo on late seizures 6 months after the start of treatment in moderate-to-severe TBI in participants aged 14 and older (1 study; *n* = 498) [25].

#### 3.2.2. Moderate Quality of Evidence

Phenytoin likely resulted in no changes in late seizures 6 to 24 months after the start of the treatment relative to other antiepileptic drugs in moderate-to-severe TBI (2 studies; *n* = 378) [25].

#### 3.2.3. Low Quality of Evidence

There may be minimal effect on the frequency of early seizures (7 days) for neuroprotective agents compared to placebo, (moderate-to-severe TBI, 1 study, *n* = 499) [25]. Antiepileptic interventions compared with placebo may reduce the frequency of early seizures (moderate-to-severe, 5 studies, *n*=987) [25]. Neuroprotective agents versus other antiepileptic drugs may have minimal effect on adverse events (moderate-to-severe TBI, 2 studies, *n* = 431) [25].

#### 3.2.4. Very Low Quality of Evidence

A review comparing baclofen 50 µg versus saline placebo included one study (*n* = 11) and examined the effects on spasticity (6 h), and adverse events [28]. The findings could not be extracted since they were not reported in the randomized control trial. The efficacy and safety of the intervention remain thereby unclear.

A review evaluated the efficacy of botulinum toxin A × 1 dose (500/1000 U) or botulinum toxin A × 1 dose of 200 U + serial casting versus placebo on spasticity (4–12 weeks post treatment), and adverse events (2 studies; *n* = 47) [28]. No statistically significant differences were detected between groups and the quality of evidence was rated very low. This hindered the ability to ascertain the true treatment effects of either intervention.

Evaluating 1029 participants and six studies, one CSR examined the difference in effects on late seizure occurrence (3 to 24 months after the start of the treatment) comparing between antiepileptic medications and placebo [25]. No significant differences were found for either outcome. The comparison was judged to provide very low quality of evidence, which indicates that the effects of antiepileptic interventions on these two outcomes remain uncertain.

In a total sample of 67 participants and one study, the reviewers found a significant difference in depression level between the repetitive transcranial magnetic stimulation and repetitive transcranial magnetic stimulation plus antidepressant groups (TBI severity unspecified) [24]. While the treatment effect was in favor of the comparator, repetitive transcranial magnetic stimulation plus tricyclic antidepressants, the true treatment effect remains uncertain due to the very low quality of evidence.

## 4. Discussion

This overview summarizes evidence on the effects of non-pharmacological and pharmacological interventions for any level of TBI severity, and reports the challenges identified in TBI research that are critical for further developing the integration and augmentation of rehabilitation services.

Amongst the options for non-pharmacological interventions, hospital-based cognitive rehabilitation and cognitive didactic therapy likely produce minimal or no changes in the return-to-work rate (moderate certainty evidence). These findings agree with published reports in the literature on neurocognitive status and the return-to-work rates, ref. [29,30,31] which maintain that favorable outcomes are facilitated by the inclusion of multidisciplinary/interdisciplinary rehabilitation services, and not by a monotherapy approach, such as cognitive rehabilitation or cognitive training alone [32,33]. Executive functions, especially sequencing and inhibitory control, are necessary to perform well at work and their status predicts the return-to-work rate following TBI [29]. Ensuring that available cognitive interventions and cognitive strategy training lead to improvements in cognitive functioning and are properly integrated in the rehabilitation management are crucial for increasing return-to-work rates, as well as improving life satisfaction and the wellbeing of individuals with TBI and their families.

The low-certainty of evidence found in acupuncture, splint therapy, and exercise of large muscle groups prevented us from ascertaining the role of these interventions on Glasgow Coma Scale scores, spasticity, and cardiorespiratory fitness, respectively. With respect to acupuncture, the lack of information on the etiology of the TBI from three of the four RCTs prevented us from determining whether the results are equally applicable to acquired brain injury, traumatic brain injury, and non-traumatic brain injury cases. Likewise, there is insufficient quality of evidence to support the roles of cognitive therapeutic approaches as monotherapy in improving community integration, depression, and activities of daily living (very low certainty evidence).

Amongst the pharmacological interventions used to reduce the number and frequency of late-seizure episodes (i.e., 6 months after the start of treatment; high-quality evidence), neuroprotective agents produced little to no difference on the frequency of late-seizures (high-quality evidence) and minimal differences on early seizures (low-quality evidence). The anti-convulsant drug, phenytoin, for example, appeared to have little effect on the number and frequency of late seizures (moderate quality evidence) and little to no effect on early-seizure events (low quality evidence). This finding aligns with current guidelines that support the use of phenytoin to treat early seizures or active seizures, but not late seizures [34].

Our evidence mapping shows that other antiepileptic drugs do not reduce the number and frequency of late seizure events. The literature primarily focuses on early seizures, and data on late seizures after TBI are limited. Discussions of study results typically note that no evidence supports the use of neuroprotective agents and antiepileptic drugs for late seizures, mainly due to the differences observed in studies on pathogenesis of early seizures in post-traumatic epilepsy [34,35]. This feature of post-TBI care warrants further attention since late seizure episodes may impair otherwise positive neurological and rehabilitation outcomes [36].

For the remaining two pharmacological interventions (botulinum toxin A × 1 dose (500/1000 U) or botulinum toxin A × 1 dose of 200 U + serial casting; intrathecal baclofen 50 μg), uncertainty of their effects on spasticity and adverse events remain, as the quality of evidence for these two therapies has been assessed as very low [28].

The absence and/or low quality of evidence for pharmacological interventions to reduce early- and late-seizure frequency, and improve spasticity, may be associated in part with the following situations: (1) research challenges exacerbated by the narrow window for effective intervention; (2) the inability of candidate medications to cross the blood–brain barrier; and (3) possible delays and ethical issues encountered when patients are unable to provide consent [37]. These difficulties are exacerbated among pediatric groups [38], which may explain the limited results for pediatric patients with TBI among the CSRs that analyzed pharmacological interventions.

The low to very- low quality evidence found is in accordance with past reviews that focused on clinical practice guidelines for TBI [39,40], which stressed the persistent paucity of quality evidence and the major gaps between the bench and the bedside in the context of rehabilitation interventions associated with both methodological issues and clinical complexity. The reviewers stated that few published trials examined rehabilitation outcomes, such as cognitive and physical function, with the majority of studies targeting symptom management or reduction [39,40].

For non-pharmacological trials, the primary issues concerned the number of studies and the small sample sizes (cumulative <500 participants), which affected the estimated effect sizes, heterogeneity among the respondents, and the imprecision of the results (i.e., wide 95% confidence intervals). Similar to pharmacological trials, some studies showed a lack of clarity regarding random sequence generation, blinding, and allocation concealment.

Overall, our evidence map shows that no prominent advances were reported in any of the CSRs, confirming the concerns expressed a decade ago by Maas et al. [41], who observed that randomized control trials (RCTs) fail to showcase significant recovery trajectories when assessing the effectiveness of interventions on TBI populations. Other study designs (e.g., observational) could provide additional insights when conducting systematic reviews for patients with TBI.

The landscape displayed by this evidence map places strong emphasis on the need to prioritize and augment rehabilitation research efforts for patients with TBI. Hence, we reiterate four priorities for bolstering the quality of evidence associated with rehabilitation outcomes: (1) revisit the recruitment and consent process and preserve ethical standards; (2) increase efforts and funding to support trials that examine functioning (i.e., cognitive, physical, and emotional); (3) consider multi-site recruitment options to increase participant diversity and sample sizes; (4) clearly identify the etiology of brain injury or offer disaggregate data in studies with mixed brain injury populations; and (5) promote the transparent reporting of adverse events, if applicable.

### Strengths and Limitations

Evidence maps represent a novel approach that can be employed to detect broader issues, lead to research synthesis, and guide researchers in formulating both future research and studies with a narrower focus [42,43]. Evidence maps have been especially helpful in visualizing research contexts and appreciating how a specific focus fits into the broader research field [44]. In the case presented here, the evidence map aids in understanding how TBI research fits within the context of clinical research and where it stands overall in the field of rehabilitation. 

A limitation that requires some discussion pertains to the search strategy. This overview exclusively analyzed systematic reviews published in the Cochrane library, which may have limited the inclusion of other high-quality systematic reviews on TBI. Nevertheless, Cochrane suggests this approach to preserve consistency in the results of the overview since the included works follow the same methodological standard [45].

We acknowledge that the evidence map developed for TBI is unable to address specific questions or nuances regarding the effectiveness of rehabilitation interventions in individuals with TBI.

Despite its limitations, the evidence map we have constructed disseminates evidence from existing literature findings on TBI, draws attention to the current challenges faced by researchers, and can provide an effective tool in guiding future research efforts and policymaking.

## 5. Conclusions

This work clarifies the need to expand research efforts in the context of TBI and clinical rehabilitation research to augment clinical applicability. In general, patients receiving rehabilitation services display a broad range of deficits and needs, which is particularly apparent among patients with TBI. Currently, the efficacy and safety of non-pharmacological and pharmacological interventions that are able to meet the needs of individuals with TBI remain uncertain, jeopardizing the clinical applicability of potentially effective interventions. To address the challenges experienced in clinical rehabilitation research, increasing the number of clinical and non-clinical trials performed that reflect sound methodology remains a priority.

## Figures and Tables

**Table 1:** Characteristics of included systematic reviews.

Author (Year)	Population	Primary Outcome	Outcome Measure	Intervention	Comparator	Effect	Quality
Hassett et al., 2017 [26]	People with TBI; any age and sex	Cardiorespiratory fitness	Submaximal incremental cycle ergometer test	Exercise using large muscle	Usual care, a non-exercise intervention, or no intervention	Favor intervention	Low
Kumar (2017) [27]	Adults (≥16 years); any sex; any severity	Return to work	Attainment of work within 14 weeks (medium-term) of initiating intervention	Cognitive rehabilitation therapy	No treatment	None	Very low
		Community integration	Sydney Psychosocial Reintegration Scale (self-reported)	Cognitive rehabilitation therapy	No treatment	None	Low
		Return to work	Return to work status Follow-up: 6 months (medium-term)	Cognitive rehabilitation therapy	Conventional therapy	None	Low
		Independence in activities of daily living	Functional independence measure, with 18 items in basic and psychosocial functional activities	Cognitive rehabilitation therapy	Conventional therapy	None	Very low
		Community integration	Community integration questionnaire	Cognitive rehabilitation therapy	Conventional therapy	None	Low
		Return to work	Return to work status Follow-up: 24 months (long-term)	Hospital-based cognitive rehabilitation therapy	Home programme	None	Moderate
		Return to work	Return to work status follow-up: 1 year (medium-term)	Cognitive didactic therapy	Functional experiential therapy	None	Moderate
		Independence in activities of daily living	Structured interview follow-up: 1 year (medium-term)	Cognitive didactic therapy	Functional experiential therapy	None	Low
Synnot (2017) [28]	Children and adults who had skeletal muscle spasticity post injury. Any severity	Spasticity at up to 6 h after treatment	Ashworth Scale, 0-,with a higher score indicating greater spasticity	Intrathecal baclofen 50 μg (injected into the lumbar spine)	Saline placebo	Not reported	Very low
		Adverse events		Intrathecal baclofen 50 μg (injected into the lumbar spine)	Saline placebo	Not reported	Very low
		Spasticity at 4–12 weeks	Modified Ashworth scale, 0–5, at 12 weeks and Tardieu scale, 0–5, at 4 weeks	Botulinum toxin A × 1 dose (500/1000 U) or botulinum toxin A × 1 dose of 200 U + serial casting	Placebo (±casting)	Uncertain	Very low
		Adverse events		Botulinum toxin A × 1 dose (500/ 1000 U) or botulinum toxin A × 1 dose of 200 U + serial casting	Placebo (±casting)	Uncertain	Very low
		Spasticity at up to 6 h after treatment	Modified Ashworth scale, 0–4, with a higher score indicating greater spasticity	Repositioning splints equipped with participant-specific pseudoelastic hinges	Traditional splints with fixed angle braces	Uncertain	Very low
		Adverse events		Repositioning splints equipped with participant-specific pseudoelastic hinges	Traditional splints with fixed angle braces	Uncertain	Very low
Gertler (2015) [24]	Children and adults with depression after TBI; any severity	Depression	Beck depression inventory-II, Hamilton Rating Scale for Depression, and Hospital Anxiety and Depression Scale; higher score means more depressed	Cognitive behavioral therapy	Wait-list control	None	Very low
		Depression	Beck Depression Inventory; higher score means more depressed	Cognitive behavioral therapy	Supportive psychotherapy	None	Very Low
		Depression	Hamilton Rating Scale for Depression; higher score means more depressed	Repetitive transcranial magnetic stimulation	Repetitive transcranial magnetic stimulation plus tricyclic antidepressant	Favor control	Very low
		Depression	Beck Depression Inventory; higher score means more depression	Supervised exercises	Exercise as usual	None	Low
Thompson (2015) [25]	People with TBI who received prophylactic treatment with antiepileptic drugs or neuroprotective agents. Any age; any severity; acute	Early seizures Follow-up: 5–7 days	Count of Events	Antiepileptic drugs	Placebo or standard care	Favor intervention	Low
		Late seizures Follow-up: 3–24 months	Count of Events	Antiepileptic drugs	Placebo or standard care	None	Very low
		Early seizure Follow-up: 7 days	Count of Events	Neuroprotective agents	Placebo	None	Low
		Late seizure Follow-up: 6 months	Count of Events	Neuroprotective agents	Placebo	None	High
		Early seizure Follow up: 7 days	Count of Events	Phenytoin	Other antiepileptic drugs	None	Low
		Late seizure Follow up: 6 months to 2 years	Count of Events	Phenytoin	Other antiepileptic drugs	None	Moderate
Wong (2013) [23]	People with TBI. Any age, sex, and severity	Post-treatment Modified Barthel Index-1 month post-treatment	Barthel index	Electro-acupuncture plus rehabilitation training	Rehabilitation training	Favor intervention	Low
		Post-treatment Modified Barthel Index-3 months post-treatment	Barthel index	Electro-acupuncture plus rehabilitation training	Rehabilitation training	Favor control	Low
		Post-treatment Fugl-Meyer assessment-1 month post-treatment	Fugl-Meyer Assessment	Electro-acupuncture plus rehabilitation training	Rehabilitation training	Favor intervention	Low
		Post-treatment Fugl-Meyer assessment-3 months post-treatment	Fugl-Meyer Assessment	Electro-acupuncture plus rehabilitation training	Rehabilitation training	Favor intervention	Low
		Post-treatment Glasgow Outcome score	Glasgow Outcome Scale	Needle acupuncture plus conventional medical intervention	Conventional medical intervention	Favor intervention	Low
		Post-treatment Glasgow Coma score	Glasgow Coma Scale	Needle acupuncture plus conventional medical intervention	Conventional medical intervention	Favor intervention	Low
		Frequency of normal post-treatment Glasgow Outcome score	Glasgow Outcome Scale	Electro-acupuncture plus conventional medical intervention	Conventional medical intervention	Favor intervention	Low
		Mortality		Electro-acupuncture plus conventional medical intervention	Conventional medical intervention	None	Low
		Frequency of post-treatment Barthel index above 60	Barthel index	Electro-acupuncture plus hyperbaric oxygen and rehabilitation training	Hyperbaric oxygen and rehabilitation training	Favor intervention	Low
		Frequency of post-treatment Barthel index above 40	Barthel index	Electro-acupuncture plus hyperbaric oxygen and rehabilitation training	Hyperbaric oxygen and rehabilitation training	None	Low

Abbreviation: TBI = traumatic brain injury.

**Table 2:** AMSTAR 2 Quality Assessment of Cochrane Systematic Reviews.

	Hassett 2017 [26]	Kumar 2017 [27]	Synnot 2017 [28]	Gertler 2015 [24]	Thompson 2015 [25]	Wong 2013 [23]
(1) Did the research questions and inclusion criteria for the review include the components of PICO?	Y	Y	Y	Y	Y	Y
(2) Did the report of the review contain an explicit statement that the review methods were established prior to the conduct of the review and did the report justify any significant deviations from the protocol?	Y	Y	Y	Y	Y	Y
(3) Did the review authors explain their selection of the study designs for inclusion in the review?	Y	Y	Y	Y	Y	Y
(4) Did the review authors use a comprehensive literature search strategy?	Y	Y	Y	Y	Y	Y
(5) Did the review authors perform study selection in duplicate?	Y	Y	Y	Y	Y	Y
(6) Did the review authors perform data extraction in duplicate?	Y	Y	Y	Y	Y	Y
(7) Did the review authors provide a list of excluded studies and justify the exclusions?	Y	Y	Y	Y	Y	Y
(8) Did the review authors describe the included studies in adequate detail?	Y	Y	Y	Y	Y	Y
(9) Did the review authors use a satisfactory technique for assessing the risk of bias (RoB) in individual studies that were included in the review?	Y	Y	Y	Y	Y	Y
(10) Did the review authors report on the sources of funding for the studies included in the review?	N	N	N	N	N	N
(11) If meta-analysis was performed did the review authors use appropriate methods for statistical combination of results?	Y	Y	Y	Y	Y	Y
(12) If meta-analysis was performed, did the review authors assess the potential impact of RoB in individual studies on the results of the meta-analysis or other evidence synthesis?	Y	Y	Y	Y	Y	Y
(13) Did the review authors account for RoB in individual studies when interpreting/discussing the results of the review?	Y	Y	Y	Y	Y	Y
(14) Did the review authors provide a satisfactory explanation for, and discussion of, any heterogeneity observed in the results of the review?	Y	Y	Y	Y	Y	Y
(15) If they performed quantitative synthesis did the review authors carry out an adequate investigation of publication bias (small study bias) and discuss its likely impact on the results of the review?	Y	Y	Y	Y	Y	Y
(16) Did the review authors report any potential sources of conflict of interest, including any funding they received for conducting the review?	Y	Y	Y	Y	Y	Y
Total	15	15	15	15	15	15

Abbreviations: Y = Yes, N = No.

**Table 3:** Evidence map of non-pharmacological interventions.

Intervention	Comparison	Outcome	GRADE			
			H	M	L	VL
Cognitive rehabilitation	No treatment	Return to work				⊗
		Community integration			⊗	
	Conventional Therapy	Return to work			⊗	
		Community integration			⊗	
		Activities of daily living				⊗
Hospital-based cognitive rehabilitation	Home-based cognitive rehabilitation	Return to work		⊗		
Cognitive didactic therapy	Functional experiential therapy	Return to work		⊗		
		Activities of daily living			⊗	
Cognitive behavioral therapy	Supportive psychotherapy	Depression				⊗
	Waitlist					⊗
Supervised exercise	Exercise as usual	Depression			⊗	
Large muscle group exercise	Usual care, non-exercise, no intervention	Cardiorespiratory fitness			✓	
Repositioning splints	Traditional splints	Spasticity				?
		Adverse events				?
Electro-acupuncture + Rehabilitation training	Rehabilitation training	Modified Barthel Index (1 mo)			✓	
		Modified Barthel Index (3 mo)			✘	
		Fugl-MeyerAssessment (1 mo)			✓	
		Fugl-MeyerAssessment (3 mo)			✓	
Needle-acupuncture + Conventional medical intervention	Conventional medical intervention	Post-Treatment Glasgow Outcome Scale			✓	
		Post-Treatment Glasgow Coma Score			✓	
Electro-acupuncture + Conventional medical intervention	Conventional medical mntervention	Frequency of Normal Glasgow Coma Score			✓	
		Mortality			⊗	
Electro-acupuncture + Hyperbanic oxygen	Rehabilitation training vs. Hyperbanic oxygen and rehabilitation training	Frequency Barthel > 60			✓	
		Frequency Barthel > 40			⊗	

High = H; M = Moderate; Low = L; VL = Very low; No effect = ⊗, Favor Intervention = ✓, Favor Comparator = ✘, Uncertain = **?**.

**Table 4:** Evidence map of pharmacological interventions.

Intervention	Comparison	Outcome	Grade			
			H	M	L	VL
Neuroprotective agents	Placebo	Early seizure			⊗	
		Late seizure (6 mo)	⊗			
Antiepileptic drugs		Early seizure			✓	
		Late seizure (3–24 mo)				⊗
Phenytoin	Antiepileptic drugs	Early seizure			⊗	
		Late seizure (6–24 mo)		⊗		
Repetitive transcranial magnetic stimulation	repetitive transcranial magnetic stimulation plus tricyclic antidepressants	Depression				✘
Baclofen 50 μg	Saline placebo	Spasticity				NR
		Adverse events				NR
Botolinum toxin A × 1 dose (500/1000 U) or botolinum toxin A × 1 dose 200 U+	Placebo	Spasticity				?
		Adverse events				?

Abbreviations: High = H; M = Moderate; Low = L; VL = Very low; No effect = ⊗; Favor Intervention = ✓; Favor Comparator = ✘; Uncertain = **?**; Not reported = NR.

## Data Availability

Not applicable.
